# 

**Published:** 1919-03-01

**Authors:** 


### Demobilisation of Doctors and Nurses.

CAPTAIN GUEST informed Mr. F. Roberts in the House of Commons that since the armistice 1,502 temporary commissioned officers of the R.A.M.C. have been demobilised and returned to civil life. The work of medical officers is centralised to avoid wastage, but the numbers released must be governed by the total number of all ranks released. The present system of demanding early demobilisation of individual officers retards the process, as in many cases substitutes have to be provided. Captain Guest, answering a further question by Mr. Lyle, said that every effort is being made to release as many doctors and nurses from the Army as can be spared.

SEAMEN'S HOSPITAL SOCIETY.—Annual Court of Governors at Prince's Restaurant, Piccadilly, Friday, March 7, 3 P.M., Admiral Sir David Beatty, G.C.B., in the chair, supported by Admiral W. S. Sims, U.S.N., Sir Kenneth Anderson, K.C.M.G., Admiral Sir Fredk. Sturdee, Bt., K.C.B., and others. Tea, 4.30.

### The College of Nursing (Limited by Guarantee).

### LONDON CENTRE.

(Hon. Secretary, Miss Biggar, St. Thomas's Hospital, S.E. 1.)

A concert for members of the Centre is to be given on Monday, March 3, at 8 p.m., in the College of Ambulance, 3 Vere Street. An attractive programme has been arranged.

### DERBY CENTRE.

(Secretary, Miss Haines, Children's Hospital, Derby.)

The last post-graduate lecture of the session in connection with the College of Nursing will be given at the Derbyshire Royal Infirmary on Tuesday next, March 4. The subject will be "Clinical Bacteriology," the lecturer being Dr. Sheila Ross, M.D. The preliminary lecture on the same subject last week was interesting and instructive. The coming election of the College of Nursing will be discussed at the next general meeting, March 13, and it is hoped all members will make an effort to attend.

### LEEDS CENTRE.

(Secretary, Miss Lindall, Women's and Children's Hospital.)

The next meeting will be held at the Leeds General Infirmary Clinical Theatre on Thursday, March 6, at 6 p.m., when Miss Grier, Acting Head, Economics Department, University of Leeds, will give a lecture on "Modern Problems in Olden Times." All nurses are cordially invited. Fee, 1s. each lecture to non-members.

